# Enhanced Recovery after an Innovative Percutaneous Endoscopic Transforaminal Lumbar Interbody Fusion for the Treatment of Lumbar Spinal Stenosis: A Prospective Observational Study

**DOI:** 10.1155/2021/7921662

**Published:** 2021-12-20

**Authors:** Peng Yin, Haifeng Gao, Lijin Zhou, Daming Pang, Yong Hai, Jincai Yang

**Affiliations:** Department of Orthopaedics, Beijing Chao-Yang Hospital, China Capital Medical, Beijing, China

## Abstract

**Background:**

The objective of this study was to investigate the enhanced recovery clinical effects of an innovative percutaneous endoscopic transforaminal lumbar interbody fusion (PE-TLIF) for the treatment of patients with LSS and degenerative instability.

**Methods:**

From January 2019 to March 2020, 51 patients with single-segment LSS and degenerative instability were prospectively included in our study (ChiCTR1900020679). The Oswestry Disability Index (ODI), the visual analogue scale (VAS) on lumbar and leg pain (VAS-LBP and VAS-LP), serum creatine kinase (CK), the peak intensity of sulphur hexafluoride microbubble contrast agent (PI), and the maximal cross-sectional area of multifidus muscle (Max-CSA) around the surgical incision were assessed preoperatively, postoperatively, and at regular follow-up.

**Results:**

All patients were followed up. The mean postoperative bedridden time was 20.45 ± 2.66 hours. The ODI, VAS-LBP, and VAS-LP were improved significantly after operation compared to these data before operation in all the patients (*P* < 0.05). The CK at 1 day after operation was higher compared to the data before the operation (*P* < 0.05), and there was no significant difference on CK at 1 week after operation (*P* > 0.05). The PI at 1 week after operation was higher compared to this item before operation (*P* < 0.05), and there was no significant difference on PI at 1 month or 3 months after operation (*P* > 0.05). The Max-CSA at 1 week after operation was higher compared to this item before the operation (*P* < 0.05), and there was no significant difference in Max-CSA at 1 month or 3 months after operation compared with before the operation (*P* > 0.05).

**Conclusions:**

Our results and systematic review presented the innovative PE-TLIF technique could obtain satisfactory and effective outcomes for the treatment of patients with LSS and degenerative instability. Our PE-TLIF technique also had the ability to decrease the MF injury and obtain an enhanced recovery.

## 1. Introduction

Lumbar spinal stenosis (LSS) is the most common type of lumbar degenerative disease for people with low back pain [1]. Several patients need to receive operation treatment due to the inefficiency of conservative treatment to alleviate the severe pain [2]. Conventional posterior lumbar fusion surgery is considered as the standard operation for patients with LSS [3]. However, extensive striping and retraction of muscle and other soft tissue usually caused some approach-related complications, especially postoperative tremendous back pain [4]; meanwhile, postoperative bedridden time was always extended.

Some studies showed that paravertebral muscle atrophy was clinically related to low back pain [5, 6]. The multifidus muscle (MF) is an important structure in paravertebral muscles, and MF atrophy is considered to be associated with low back pain [7]. Recently, it was reported that the volume of the multifidus muscle was dramatically decreased after the open posterior lumbar fusion [8]. Hence, it is necessary to perform a minimally invasive lumbar surgery to decrease the injury of the MF and shorten the postoperative rehabilitation period.

Endoscopic lumbar fusion techniques have gradually gained popularity in the past several years, and we have also developed an innovative minimally invasive surgery named percutaneous endoscopic transforaminal lumbar interbody fusion (PE-TLIF) [9].The preliminary results of our technique were satisfactory, but the injury of the MF and enhanced recovery were not evaluated in our previous study. Hence, we conducted a prospective observation study on the MF injury via contrast-enhanced ultrasonography after PE-TLIF for the treatment of patients with LSS to provide evidence of obtaining an enhanced recovery. Meanwhile, we further investigated the clinical effects of PE-TLIF in order to provide good evidence for clinical practice.

## 2. Material and Methods

From January 2019 to March 2020, 51 patients with single-segment LSS and degenerative instability were included in our study (ChiCTR1900020679). The eligible criteria were as follows: (1) patients with LSS and degenerative instability on L4/5 level; (2) patients treated by PE-TLIF; (3) no lumbar surgery history; (4) no obvious multifidus muscle injury; and (5) no lumbar deformity. The exclusion criteria were as follows: (1) patients were unable to finish the follow-up; (2) patients with other comorbidity could affect the lumbar fusion; and (3) patients with other comorbidity could affect the serum creatine kinase. All patients were told all possible results during the study and signed written consent before the operation. The study was approved by the institutional review board of Beijing Chaoyang Hospital.

**Figure 1:**
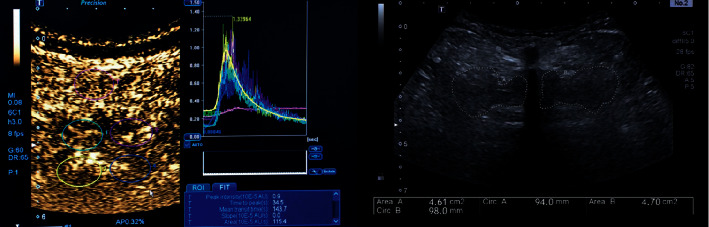
Contrast-enhanced ultrasonography was used to demonstrate the blood perfusion of the multifidus muscle microcirculation.

Appropriate perioperative assessments were conducted for all patients before the operation. 51 patients were operated by the PE-TLIF technique. There were 37 females and 14 males, and the average age was 58.98 ± 8.64 years. Operative level was L4/5. Operation time, intraoperative bleeding volume, postoperative drainage volume, and postoperative bedridden time were recorded. The intervertebral fusion was evaluated via the Bridwell criteria at 6 months after the operation. The Oswestry Disability Index (ODI), visual analogue scale (VAS) on lumbar and leg pain (VAS-LBP and VAS-LP), serum creatine kinase (CK), the peak intensity of sulphur hexafluoride microbubble contrast agent (PI), and the maximal cross-sectional area of multifidus muscle (Max-CSA) around the surgical incision were calculated via contrast-enhanced ultrasonography at 1 week, 1 month, 3 months, and then at final follow-up (Figure 1).

### 2.1. Surgical Techniques

**Figure 2:**
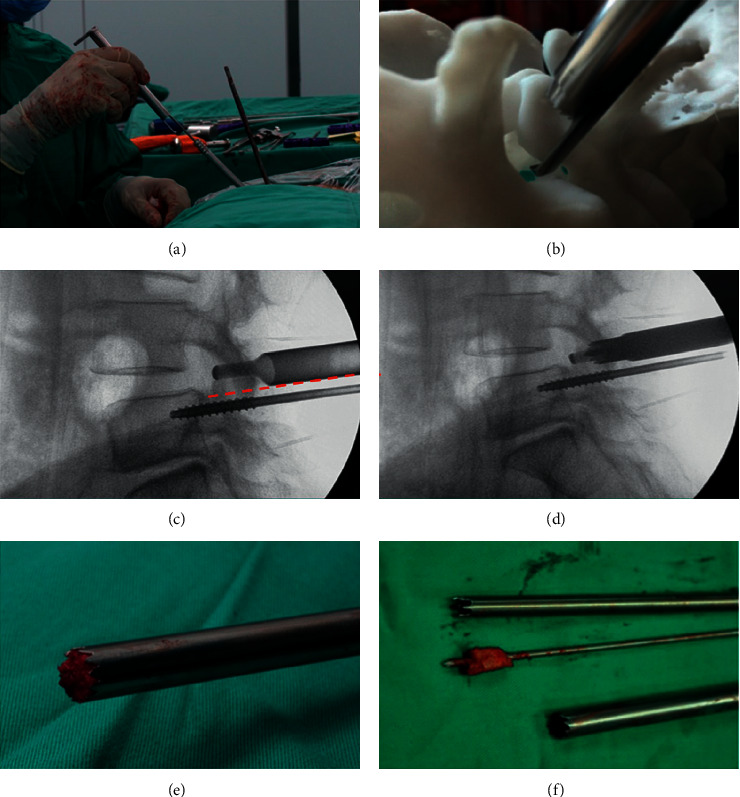
Resection method of the superior articular process. The hook-shaped protective sleeve clings to the lateral periosteum of the superior articular process, reaches the ventral side of the articular process, protects the exiting nerve root and can control the cutting depth of the trephine at the same time, protects the dura mater and nerve root, and rotates the trephine to remove the superior articular process.

**Figure 3:**
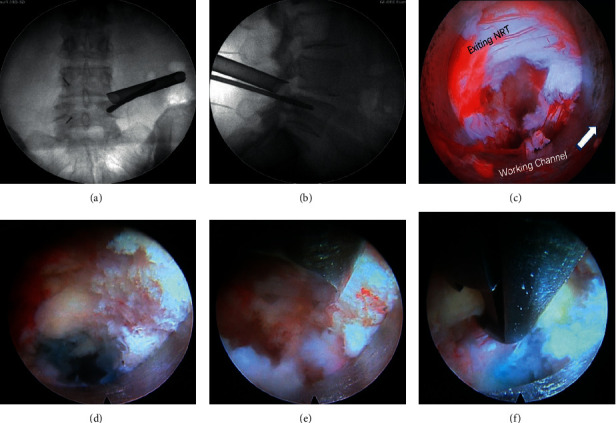
Full-endoscopic decompression. (a, b) The optimal position of the working channel is confirmed under the fluoroscopy of the C-arm. In anteroposterior fluoroscopy, the front end of the working channel reaches the outer edge of the vertebral body. In lateral fluoroscopy, the front end of the working channel reaches the posterior edge of the vertebral body (image from the other patient). (c) Place the endoscope, the blue disc is easy to find and the nerve root should be protected carefully. Confirm the exiting nerve root under endoscopy and protect the nerve root by rotating the tongue-shaped working channel. Remove the surrounding nucleus pulposus tissue and relieve the nerve root. (d) The stump of the superior articular process after trephine cutting. (e) The stump is dealt with an osteotome under an endoscope to ensure complete resection of the superior articular process until the upper wall of the pedicle is exposed. (f) The laminar rongeur is used to remove the hyperplastic ligamentum flavum and reveal the transversing nerve roots under the endoscope.

**Figure 4:**
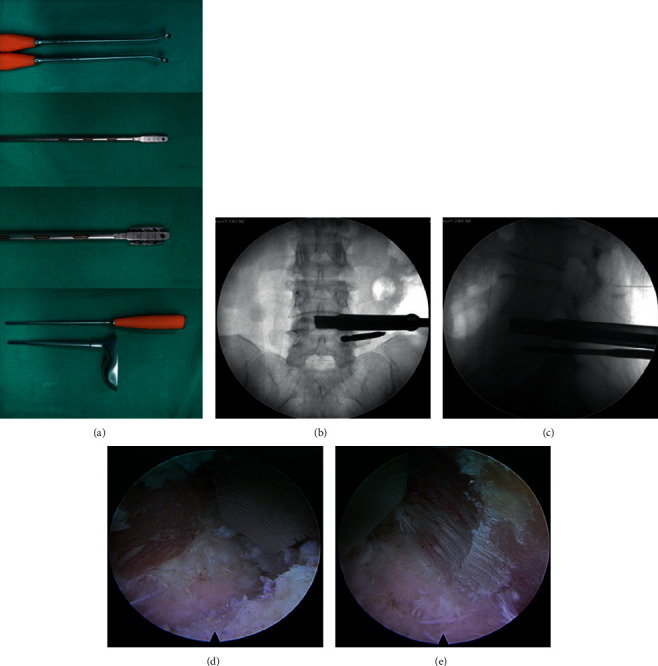
Endplate preparation. (a) From up to down of the picture, the 15-degree angle endplate curette, the width adjustable reamer, and the bone grafting device. (b, c) The reamer is used to prepare the cartilage endplate to adequately expose the bony endplate. (d, e) The intervertebral space is fully prepared and the appearance of exudation from the bone endplate is good, the bony endplate is fully exposed. The width of the bone graft bed is in a fan-shaped area greater than 13 mm at the proximal section and 15 mm at the distal end.

The patients were in a prone position. General anesthesia or low-dose epidural anesthesia combined with local anesthesia was applied during the PE-TLIF surgery. The C-arm fluoroscope was employed to confirm the surgical lumbar segment. The primary guide pin was inserted into the pedicle of the symptomatic side, and a specially designed SAP guider was used to put the secondary guide pin into fixation at the superior articular process (SAP). Then, dilating cannulas were inserted gradually through the secondary guide pin. A hook-shaped front of the cannula was applied to ensure the majority of SAP was excised safely by trepan (Figure 2). The endoscope system was connected after the working channel was placed through Kambin's triangle. The canal and nerve root were decompressed with the surveillance of the endoscopy (Figure 3), and then the complete endplate preparation was performed (Figure 4). The acceptable extent of endplate preparation under endoscopy was the appearance of hemic exudation from the bone endplate. An expandable cage (Shanghai Reach Medical Instrument Co., Ltd, Shanghai, China) with iliac bone autograft was then inserted through the working channel. Iliac bone autografting and adequate bone graft size (≥5 mm^3^ per intervertebral space). The nerve root was confirmed to be totally relieved via endoscopy once again. Finally, four pedicle screws and two rods were inserted percutaneously. The active bleeding was stopped under the surveillance of endoscopy, and the incisions were sutured. More details on the PE-TLIF technique were described in our previous study [9].

### 2.2. Statistical Analysis

The data were analyzed by SPSS 17.0 software with chi-square and Fisher's exact test for nominal data and an independent *t*-test in continuous data. A statistically significant difference was determined when *P* < 0.05.

## 3. Results

All patients underwent PE-TLIF surgery successfully. The mean operation time was 202.65 ± 27.52 minutes. The average intraoperative bleeding volume was 125.20 ± 40.41 ml. The average incision length was 8.54 ± 2.22 cm. The mean postoperative bedridden time was 20.45 ± 2.66 hours.

**Figure 5:**
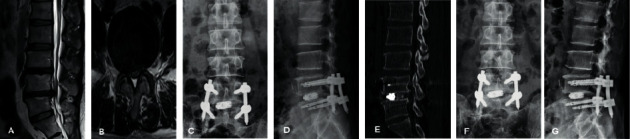
A 65-year-old female patient was diagnosed as lumbar spinal stenosis with degenerative instability and was treated by PE-TLIF. (a, b) Preoperative MRI demonstrated a lumbar spinal stenosis at L4/5. (c, d) A good implantation position was presented through X-ray images taken 7 days after the operation. (e) A standard lumbar fusion was presented via CT scan image at 6 months after the operation. (f, g) X-ray images demonstrated a good implantation position at final follow-up.

All patients were followed up, and the average follow-up period was 18.70 ± 4.54 months. The ODI at 3 months after the operation and at final follow-up were improved significantly compared to the data before operation in all patients (*P* < 0.05). The VAS-LBP and VAS-LP at 1 week, 3 months, and 6 months after the operation and at final follow-up improved significantly compared to these data before operation in all the patients (*P* < 0.05) (Table 1 and Figure 5).

The CK at 1 day after the operation was higher compared to the data before the operation (*P* < 0.05), and there was no significant difference in CK at 1 week after the operation (*P* > 0.05). The PI at 1 week after the operation was higher compared to this item before the operation (*P* < 0.05), and there was no significant difference in PI at 1 month, 3 months after the operation, and also at final follow-up compared to this item before the operation (*P* > 0.05). The Max-CSA at 1 week after the operation was higher compared to this item before the operation (*P* < 0.05), and there was no significant difference in Max-CSA at 1 month 3 months after the operation, and also at final follow-up compared with this item before the operation (*P* > 0.05). More details are listed in Table 2.

All the patients finished the intervertebral fusion at 6 months after the operation. According to the Bridwell criteria, 23 patients were rated as Grade 1, 24 patients as Grade II, and 4 patients as Grade III. One patient suffered temporary knee tendon hyperreflexia after surgery and recovered within 24 hours after surgery.

## 4. Discussion

This is the first prospective observation study on the MF injury via contrast-enhanced ultrasonography after PE-TLIF for the treatment of patients with LSS and degenerative instability at present. Our present results showed that the MF injury in the PE-TLIF technique could be recovered at 1 month after the operation, and the CK at 1 week after the operation was restored to the status before the surgery. The ODI and VAS were significantly improved for all the patients via the PE-TLIF surgery. The mean postoperative bedridden time was less than 24 hours.

The MF plays an important role in the preservation of lumbar segmental stability and stiffness [10]. The MF is the most medial component of the lumbar paraspinal muscles and is only innervated by the medial branch of the dorsal ramus, without intersegmental nerve supply [11]. Hence, iatrogenic denervation of the MF usually occurs during the dissection and retraction in conventional posterior lumbar fusion surgery. Some researchers believed that postoperative muscle atrophy was related to iatrogenic denervation of the paraspinal muscles during the operation [12]. The MF atrophy was mostly associated with postoperative low back pain [8]. Therefore, most spine surgeons demonstrate that decreasing the MF injury is vital for the improvement of the postoperative functional outcomes and shortening the rehabilitation period. Hence, minimizing the MF injury during the surgery gradually became a pursuing goal.

Minimally invasive spine surgeries (MISS) have gained popularity to decrease the muscle-related injury on the conventional open surgeries. Schwender et al. first described the minimally invasive transforaminal lumbar interbody fusion (MIS-TLIF), and the technique showed the potential advantages in the aspect of soft tissue injury over conventional open techniques [13]. However, the placement of screws in the MIS-TLIF technique is very similar to conventional open surgeries, so the medial branch of the dorsal ramus is usually injured, which could increase the possibility of the MF atrophy. Regev et al. believed that percutaneous screw placement was able to decrease the indirect injury of the medial branch nerve from 84% to 20% [14].Therefore, more and more surgeons have begun to attempt endoscopic lumbar fusion techniques with percutaneous screw fixation for lumbar degenerative diseases.

In our study, we developed an innovative endoscopic fusion technique named PE-TLIF, and the initial clinical results were favorable [9]. Postoperative serum CK level was considered as a marker of intraoperative related muscle injury [15]. The CK level was recovered to the preoperative level in all the patients, which was consistent with the previous report [16]. The minimally invasive technique could reduce the injury extent of the muscle. CSA was rated as a valuable indicator of MF injury. Some studies showed that the postoperative CSA of the MF was significantly smaller than the preoperative status [16, 17]. In our study, there was no difference in the Max-CSA via contrast-enhanced ultrasonography between the preoperative level and the level at 1 month after the PE-TLIF technique. We also investigated the PI through contrast-enhanced ultrasonography, and the PI was restored to the preoperative level at 1 month after the PE-TLIF technique. The CK, Max-CSA, and PI were higher than the preoperative items in the early postoperative stage, which was mostly associated with the hemorrhage and edema. Our technique made decisive technical improvements, and the approach could not directly injure the MF. We also chose the percutaneous method to place the screws to avoid injury of the medial branch nerve. Hence, the PE-TLIF technique could minimize the injury of the MF. All the patients obtained satisfactory clinical effects via the evaluation of ODI and VAS, and the postoperative lumbar pain was significantly decreased through our technique. The mean postoperative bedridden time was less than 24 hours in all the patients.

To obtain a comprehensive understanding on endoscopic lumbar interbody fusion, we did a systematic review on endoscopic lumbar interbody fusion for the treatment of lumbar degenerative diseases until December 2020. Finally, 20 studies were included in our present systematic review (SR) [9, 18–36]. Most studies presented that the endoscopic lumbar fusion technique was a promising treatment for lumbar degenerative diseases, with less muscle injury and quicker rehabilitation. The fusion rate was 59.6%–100%, and the complication rate was 0%–36%. However, there were no standard operating procedures and specific indications of endoscopic lumbar interbody fusion. More details are listed in Tables 3 and 4.

To the best of our knowledge, this is the first prospective observational study on MF injury via contrast-enhanced ultrasonography after PE-TLIF for the treatment of patients with LSS and degenerative instability at present. All surgeries were performed by one senior surgeon. Several data on the characteristics of patients and clinical effects were reported in our study. The core part of our innovative technique is to excise the majority of SAP safely and effectively. We innovated a hook-shaped front of the cannula, which could be a very useful tool to excise SAP, while could protect soft tissues and nerves. The contradiction on our technique is the severe central type of LSS. However, certain limitations need to be addressed. Our study lacks the conventional control group, and the number of patients is relatively small. More prospective randomized controlled trials are needed to overcome the limitations of our study.

In conclusion, our results and SR presented the innovative PE-TLIF technique could obtain satisfactory and effective outcomes for the treatment of patients with LSS and degenerative instability. Our PE-TLIF technique also had the ability to decrease MF injury. Patients with PE-TLIF could have a quicker postoperative rehabilitation.

## Figures and Tables

**Table 1:** Comparison of indicators related to efficacy evaluation before and after PE-TLIF.

*n* = 51	VAS-LBP	VAS-LP	ODI (%)
Preoperation	7.22 ± 0.12	6.24 ± 0.18	63.28 ± 2.12
Post-7d	3.61 ± 0.16^①^	2.45 ± 0.18^①^	—
Post-1m	1.24 ± 0.07^①^	1.22 ± 0.13^①^	__
Post-3m	1.06 ± 0.11^①^	0.98 ± 0.12^①^	14.8 ± 1.10^①^
Final follow-up	0.67 ± 0.09^①^	0.41 ± 0.07^①^	9.00 ± 0.86^①^

*Note.* ①Compared with preoperation, *P* < 0.05.

**Table 2:** Comparison of indicators related to multifidus injury before and after PE-TLIF.

*n* = 51	CK(U/L)	Max-CSA (mm^2^)	PI (db)
Preoperation	83.79 ± 4.99	520.29 ± 10.27	2.68 ± 0.11
Post-1d	452.62 ± 13.50^①^	—	—
Post-7d	91.23 ± 2.71^②^	628.20 ± 11.26^①^	4.55 ± 0.17^①^
Post-1m	—	516.74 ± 7.86^②^	2.91 ± 0.06^②^
Post-3m	—	482.29 ± 8.61^②^	2.53 ± 0.06^②^
Final follow-up	—	477.42 ± 8.80^②^	2.36 ± 0.08^②^

Note: ①Compared with preoperation, *P* < 0.05; ②compared with preoperation, *P* > 0.05.

**Table 3:** Characteristics of included studies.

First author	Study no.	Year	Study design	No. of patients	Intraoperative never monitoring	Anesthesia method	Indication	Follow-up (months)
Osman	1	2012	RS	60	Yes	General anesthesia	DDD (8.3%), LSS (81.7%), and SL (10%)	12(6–25)
Jacquot	2	2013	RS	57	No	Local anesthesia	DDD (100%)	24
He	3	2015	RS	42	Yes	General anesthesia	LSS (81.0%), DSL (14.3%), and LDH (4.8%)	27.6 ± 3.8(24–36)
Morgenstern	4	2015	RS	30	Yes	General or local anesthesia	DDD (30%), SL (40%), FA (20%), IAD (6.67%), and CD (3.33%)	38 ± 17(11–67)
Wang	5	2016	RS	10	Yes	Local anesthesia	DDD (100%) and SL (60%)	12
Lee	6	2017	RS	18	No	Local anesthesia	DDD (88.9%) and SL (11.1%)	46(12–123)
Heo	7	2017	RS	69	No	General or epidural anesthesia	SL (87.0%) and LSS (13%)	13.5 ± 7.1
Zhang	8	2017	RS	17	No	General anesthesia	LSS (100%)	12
Myung	9	2018	RS	None	No	Local anesthesia	None	None
Kim	10	2018	RS	14	No	General anesthesia	LSS (57.1%) and SL (42.9%)	2
Wu	11	2018	RS	7	Yes	General anesthesia	SL (100%)	35.1 ± 3.0(31.5–38.1)
Yang	12	2019	RS	7	Yes	Epidural or local or general anesthesia	LSS (100%)	15 (12–21)
John	13	2019	RS	100	No	Local anesthesia	DDD with SL (100%)	36
Park	14	2019	RS	71	No	General anesthesia	LSS (9.9%), SL (87.3), and HNP (2.8)	12
Wu	15	2020	RS	44	No	Local anesthesia	LDH (50%) and LSS (50%)	13.2 ± 3.2
Wu	16	2020	RS	91	No	Local anesthesia	LDH or LSS with SL (100%)	20.0 ± 4.1
Jin	17	2020	RS	39	No	Epidural or local anesthesia	DLD (100%)	23.6 ± 4.9 (17–28)
Morgenstern	18	2020	RS	51	No	Epidural or local or general anesthesia	DDD (84%) and SL (31%)	27.9 ± 27
Harakuni	19	2020	RS	12	No	General anesthesia	DDD (100%)	6.2(2–10)
Zhang	20	2020	RS	1	Yes	General anesthesia	LSS (100%)	12

RS: retrospective case series; DDD: degenerative disc disease; DLD: degenerative lumbar disease; LSS: lumbar spinal stenosis; SL: spondylolisthesis; PO: previous operation; DSL: degenerative spondylolisthesis; FA: failed arthrodesis; IAD: instability after decompression; CD: chondroma.

**Table 4:** Interventions and outcomes of included studies.

Study no.	Surgical technique	Resection of articular process	Operation time (minutes)	Blood loss (ml)	Spinal decompression	Internal fixation method	Fusion rate	Complications
1	Endo-LIF	No	174 (117–251)	57.6 (30–100)	No	Bilateral PS	59.6%	8 patients RSE, 2 patients RN, and 2 patients PSC
2	PE-TLIF	No	60 ± 30	None	No	Bilateral PS	77%	8 patients RPP and 13 patients AMC
3	FE-MIS-TLIF	Yes	133.9 ± 16.1(OS) 241.3 ± 36.5 (TS)	221.8 ± 98.5 (100–550)	Yes	Bilateral PS	92.9%	2 patients PNC
4	pTLIF	Yes	120 ± 30 (A or B) 240 ± 120 (C)	None	No	Bilateral PS	None	3 patients TD and 2 patients SIP
5	E-MIS-TLIF	Yes	113.5 ± 6.3(105–120)	65 ± 38 (30–190)	Yes	Bilateral PS	None	No complications
6	PTLIF	No	77 (62–100)	None	No	Bilateral PS	88.9%	1 patient PNC, 1 patient nonunion, and 1 patient revision
7	UBE	Yes	165	85.5 ± 19.41	Yes	Bilateral PS	None	2 patients dura tear and 3 patients postoperative hematoma
8	Endo-TLIF	Yes	174 (130–235)	95 (50–200)	Yes	Bilateral PS	100%	2 patients transient nerve root paresthesia
9	FELIF	Yes	None	None	Yes	Bilateral PS	None	None
10	BE-TLIF	Yes	169 ± 10	74 ± 9	Yes	Bilateral PS	None	1 patient L5 paralysis and 1 patient dura tear
11	PELIF	Yes	167.5 ± 30.9 (135–220)	70.0 ± 24.5 (50–100)	Yes	Bilateral PS	None	No complication
12	PE-TLIF	Yes	285	117.1 (30–300)	Yes	Bilateral PS	100%	1 patient disc ruptured and 1 patient temporary knee tendon hyperreflexia
13	Endoscopic MIS-TLIF	No	1 level: 84.5 ± 21.7 2 levels: 128.1 ± 48.6	1 level: 65.4 ± 76.6 2 levels: 74.7 ± 33.6	Yes	Bilateral PS	100%	4 patients convert to general anesthesia, 2 patients cage migration, 1 patient osteomyelitis, and 1 patient endplate fracture
14	ULIF	Yes	158	None	Yes	Bilateral PS	25.9%(PF) 74.1%(DF)	3 patients dural tear, 1 patient hematoma, and 1 patient infection
15	Endo-LIF	Yes	184.3 ± 70.6 191.1 ± 32.4	38.5 ± 19.5 214.6 ± 61.6	Yes	Spinous process laminar screw	95%	EG: 3 patients low back pain, CG: 1 patient cerebrospinal fluid leakage, and 1 patient incision infection
16	MIS-TLIF	Yes	180.49 ± 35.19 164.02 ± 51.91	182.00 ± 106.19 191.30 ± 93.37	Yes	Bilateral PS	None	EG: No complication and CG: 1 patient hematoma
17	PELIF	Yes	213.8 ± 31.7 (185–324)	25.0 ± 12.6 (15–50)	Yes	Bilateral PS	100%	2 patients symptom was not relieved or even aggravated, 1 patient disc mass remnant, 1 patient misplacement of L5 pedicle screw, and 1 patient asymptomatic cage subsidence
18	pTLIF	Yes	None	None	No	Bilateral PS	None	12 patients transitory and ipsilateral dysesthesia, 2 patients transitory and ipsilateral muscle weakness, and 3 patients sacroiliac joint pain
19	PELIF	Yes	109.4 (73–160)	None	No	Bilateral PS	100%	1 patient paresthesia in both legs and 1 patient left knee pain
20	Endo-TLIF	Yes	None	None	Yes	Bilateral PS	100%	No complication

CG: control group; VAS: visual analogue scale; RMDQ: Roland–Morris Disability Questionnaire; EG: experiment group; ETD: endoscopic transforaminal decompression; LIF: lumbar interbody fusion; PPSI: percutaneous pedicle screw implantation; RSE: residual discomfort on extension; RN: residual numbness; PSC: pedicle screw-related complications; PE-TLIF: percutaneous endoscopic transforaminal lumbar interbody fusion; RPP: radicular pain with paresthesias; TS: two segments; AMC: asymptomatic migration of the cages: ODI: Oswestry Disability Index; OS: one segment; FE-MIS-TLIF: full-endoscopic minimally invasive transforaminal lumbar interbody fusion; PNC: postoperative neurological complications; PTLIF: percutaneous transforaminal lumbar interbody fusion; TD: transitory dysesthesia; SIP: sacroiliac pain; SF-36: 36-item short form health survey; UBE: unilateral biportal endoscopic technique; DT: dural tear; PEH: postoperative epidural hematoma; BE: biportal endoscopic; PF: probable fusion; DF: definite fusion.

## Data Availability

The data used to support the findings of this study are available from the corresponding author upon request.
